# Case of Intus-susception of the Small Intestine

**Published:** 1845-06

**Authors:** 


### SURGICAL SOCIETY OF IRELAND, MARCH 15, 1845.

*Case of Intus-susception of the Small Intestine.*—Dr. BENSON begged leave to present to the society a specimen of morbid anatomy which he believed to be very rare. It was the small intestine of an adult, in which four examples of intus-susception are to be observed. Dr. Benson was aware that it was not very unusual to find two, three, or more intus-susceptions in the intestines of a child, but although he had opened as many abdomens as most men, he had never met with four in one adult until now.

The patient in whom this intestine was found had been a sailor, aged 35, of intemperate habits, who had travelled much, and had been afflicted with yellow fever in the West Indies some years ago. He was admitted a short time since into the City of Dublin Hospital, with enlarged and indurated liver, and jaundice. Under treatment he was improving a good deal, until hematemesis set in, under which he sunk in thirty-six hours.

On the post-mortem examination the stomach and all the intestines, as far as the sigmoid flexure of the colon, were found full of blood, the characters of which gradually changed as it approached this part, and was here of pitchy colour and appearance. The intus-susceptions were all in the jejunum, and did not seem to cause any obstruction to the onward course of the blood, nor to be attended with any mark of inflammation in the peritoneum investing them. They had all the marks of having been very recently formed, and one of them was actually unfolded by the pupils who carelessly handled them. They were all of the usual kind—the upper part received into the lower. During life there was no symptoms to lead one to suspect their existence—no constipation—no violent colic.

Dr. Benson would ask, what was the relation between these intus-susceptions and the hæmorrhage from the gastro-intestinal mucous membrane? Could it be said that either was the cause of the other? He thought it very likely that they were caused by the blood acting as an irritant on the intestine, and giving rise to those irregular contractions and dilatations which resulted in the peculiar morbid appearance before them. Every one must have observed what severe gripings generally attend intestinal hæmorrhages. And even when blood is swallowed in epistaxis, it not unfrequently causes tormina, though in such a case the blood must have undergone changes in the stomach; how much more likely would it be to occasion these pains, when it came unchanged, and as a foreign body, in contact with a membrane unprepared for its reception!

On the other hand, he (Dr. B.) thought it very unlikely that the intus-susceptions caused the hæmorrhage in this case. No doubt hæmorrhage might arise from such a cause; the interruption to the circulation in the part by the stricture might be followed by some extravasation, but the stricture which could do that would also prevent the blood from getting on in the bowel; it would also be of longer standing, it would show more marked symptoms of colic in life, and it would not have caused so copious a discharge of blood from the stomach.

Dr. Benson concluded, therefore, that the blood in the intestines caused the irregular actions which ended in intus-susception; that the intus-susceptions occurred only a short time before the death of the patient; and that the fatal termination could in no wise be attributed to them. They were, however, he thought, deserving the attention of the society; and he would be glad to know if any member present had met with similar appearances under similar, or under any circumstances?—***Dublin Medical Press.***

